# Hepatoprotective effect of *Matricaria chamomilla* aqueous extract against 1,2-Dimethylhydrazine-induced carcinogenic hepatic damage in mice

**DOI:** 10.1016/j.heliyon.2020.e04082

**Published:** 2020-06-01

**Authors:** Salima Shebbo, Manal El Joumaa, Rawan Kawach, Jamilah Borjac

**Affiliations:** Department of Biological Sciences, Beirut Arab University, Debbieh, Lebanon

**Keywords:** Dimethylhydrazine, *Chamomile*, Liver injury, Wnt signaling, COX-2, iNOS, Biochemistry, Biological sciences, Cancer research, Hepatobiliary system, Molecular biology, Oncology, Pharmaceutical science, Microbiology, Pharmaceutical chemistry, Toxicology

## Abstract

Dimethylhydrazine (DMH) is a potent colonic and hepatic carcinogen that is metabolized into oxyradicals causing liver injury and DNA mutations. *Matricaria chamomilla* is a well-documented medicinal herb that possesses anti-inflammatory, antioxidant and antitumor activities and is commonly used to treat diverse ailments. The present study aimed to reveal the hepatoprotective effects of *Matricaria chamomilla* aqueous extract during an intermediate stage of colorectal cancer (CRC) in mice. Male Balb/c mice were divided into six groups: group A served as control, group B received chamomile extract (150 mg/Kg b.w.) orally for 12 weeks, and groups C-F received weekly intraperitoneal injections of DMH (20 mg/Kg b.w.) once a week for 12 weeks. In addition to DMH, groups D and F received chamomile during the initiation and post-initiation stages, respectively. Blood and liver samples were collected for biochemical and molecular analyses. The results showed that DMH induced hepatic injury in mice as shown by significant increase in serum aspartate aminotransferase and alanine aminotransferase. The changes in biochemical parameters were accompanied by activation of the Wnt signaling pathway leading to increased hepatocytes proliferation as well as inflammation evidenced by high levels of pro-inflammatory enzymes cyclooxygenase 2 (COX-2) and inducible nitric oxide synthase (iNOS). The results also showed potential hepatoprotective effects of chamomile extract against DMH-induced liver injury, proliferation and inflammation. Chamomile restored the biochemical and molecular parameters and this improvement was more pronounced in mice pretreated with the extract. In conclusion, chamomile extract may exert its hepatoprotective activities against DMH probably due to the antioxidant, antiproliferative and anti-inflammatory properties of its flavonoids.

## 1 Introduction

Colorectal cancer (CRC) is the third prevalently diagnosed cancer worldwide and has become a major cause of cancer-related mortality [1]. CRC develops from multistep processes that establish accumulating pre-neoplastic lesions in mucosal cells leading to cancer [2]. Due to the observed geographic differences in CRC rates, several epidemiological studies have suggested that diet strongly influences the occurrence of this disease [3]. For instance, Western diets that are rich in fat and red meat constitute major risk factors for CRC development; however, fruit, vegetables, and dietary fibers are commonly associated with a reduced risk of CRC [3,4].

1,2-Dimethylhydrazine (DMH) is a toxic environmental pollutant [5] that has been detected in tobacco [6], some mushrooms and food items [7,8] as well. DMH has been well-documented as a potential carcinogen with selective toxicity for colon and rectum in animal models [9]. Also, it is a powerful hepatocarcinogen that induces oxidative stress, hepatotoxicity, and hepatocellular carcinoma upon its metabolism in the liver [10]. In addition, its metabolites, methyldiazonium ion and a reactive carbonium ion, methylate guanines in DNA forming O6-methyl-deoxyguanosine and N7-methyl-deoxyguanosine, thus inducing genetic mutations in diverse genes such as the adenomatous polyposis coli gene (Apc) and β-catenin gene (Ctnnb1) [11]. These genes are key players of the Wnt pathway, one of the most important and conserved signaling pathways involved in colon and liver cancers [12]. Several studies showed that DMH metabolites cause missense or point mutations in Apc gene and point mutations in the Ctnnb1 gene [13, 14, 15]. In addition, DMH causes the accumulation of pro-inflammatory enzymes such as cyclooxygenase 2 (COX-2) [16] and inducible nitric oxide synthase (iNOS) [17] that play pivotal roles in inflammation and tumor growth in humans and experimental models.

Since conventional and synthetic drugs used in the treatment of diseases, including cancer, have a vast array of unfavorable side effects, there is an increasing worldwide interest in the use of traditional medicinal herbs to treat various diseases [18]. The therapeutic uses of medicinal herbs have many advantages including their safety and easy availability besides being economical and effective [19]. Chamomile, scientifically named by Linnaeus *Matricaria chamomilla* L.*,* is one of the most commonly used medicinal herbs whose extracts and standardized tea are usually prepared from the dried flowers [20]. Chamomile is a member of the daisy family (*Asteraceae*) that has been traditionally used in treating wounds, eczema, ulcers, gout, skin irritations, burns, neuralgia, rheumatic pain, hemorrhoids, diaper rash, chicken pox, ear and eye infections, and respiratory disorders [21,22]. In addition, chamomile has been used as a digestive relaxant treating various gastrointestinal disturbances including indigestion, flatulence, diarrhea, motion sickness, anorexia, nausea, and vomiting [23, 24, 25].

Chamomile contains different bioactive constituents such as the blue oil (0.24%–1.9%) containing terpenoids, α-bisabolol and chamazulene, farnesene, spiro-ether quiterpene lactones, hydroxycoumarins, glycosides, flavanoids (apigenin, luteolin, patuletin, and quercetin), coumarins (herniarin and umbelliferone), and terpenoids [26]. Chamomile is widely considered as a sleep-inducer and a mild tranquillizer [27]. Furthermore, some studies suggest that chamomile extracts possess hypoglycemic [28], hepatoprotective [29], antioxidant [30], and antitumor effects against skin, prostate, breast, ovarian, and colorectal cancer [31,32].

Recently, a study by El Joumaa *et al.* [33] revealed a chemoprotective role of aqueous chamomile extract against the DMH-induced model of CRC. In their study, the chemopreventive and antitumor effects of chamomile were mediated via downregulating the Wnt signaling pathway and mitigating inflammation in the colons of DMH-injected mice. In addition, since chemical carcinogens including DMH require metabolic activation in the liver in order to exert their mutagenic and carcinogenic effects [9], we hypothesized that chamomile extract might exert hepatoprotective effects against DMH-induced carcinogenesis. In this context, the present study was designed to provide a better understanding of the potential action of chamomile extract against DMH-induced hepatocarcinogenicity in mice.

## 2 Materials and methods

### 2.1 Chemicals

1,2-Dimethylhydrazine dihydrochloride was obtained from ACROS Organics™ (part of Thermo Fisher Scientific, NJ, USA). Phenylmethanesulfonylchloride (PMSF) was purchased from Roche Diagnostics (Risch-Rotkreuz, Switzerland). All primers were purchased from BIO-RAD® (CA, USA) except GAPDH primers which were synthesized by TIB Molbiol (Berlin, Germany). All other chemicals and reagents used were of high commercial and analytical grades.

### 2.2 Chamomile extract

Air-dried chamomile flowers of Syrian origin were purchased from a local market in Saida city, Lebanon. The taxonomic identification of this herb was performed by Dr. Salwa Mahmoud Abdul Rahman, Department of Biological Science, Faculty of Science at Beirut Arab University. Chamomile's flowers (2.5 g) were soaked in 100 mL of boiled distilled water (100 °C) and steeped at room temperature for 30 min with occasional stirring. The mixture was then filtered, aliquoted and stored at -20 °C to be used.

### 2.3 Extraction, UPLC and LC-TSQ-Endura-MS/MS analysis of polyphenols and flavonoids

The aqueous extract was filtered with 0.25 μm Millipore SPE cartridges and diluted 1:10 with LCMS grade water. The resultant crude solution was injected into a UPLC-PDA (Thermo Scientific, MA, USA) using a C18-Hypersil Gold reverse phase column to acquire a fingerprint 3D chromatogram. Gradient elution was performed with 0.1% formic acid in water/acetonitrile at a constant flow rate of 0.285 mL/min and an injection volume of 10 μL. Separation was carried out in 30 min.

A list of 50 common polyphenols and flavonoids (Table 1) was formulated based on a literature review on the constituents of chamomile and culinary herbs [34]. The 50 compounds were then analyzed via direct injection into a UPLC-TSQ-Endura triple Quadruple mass spectrometer (Thermo Scientific, MA, USA) equipped with an ESI source operating in both positive and negative ion mode. In positive ionization mode, the mobile phase used was 10% methanol:water in formic acid at a flow rate of 250 μL/min while in negative ionization mode the same mobile phase was used but without formic acid. The detection and qualitative analysis was carried out based on MRM transitions reported by Vallverdú-Queralt *et al.* [34] and by PubChem Mass Spectral Data (National Center for Biotechnology information, URL: https://www.ncbi.nlm.nih.gov/pccompound).

**Table 1:** List of polyphenols and flavonoids screened for via LC-MS/MS.

	Compound
1	alpha-Bisabolol
2	Chamazulene
3	Methyl angelate
4	Angelic acid
5	Isobutyl angelate
6	Farnesene
7	alpha-Pinene
8	Nobilin
9	3-Epinobilin
10	Bisabolol oxide A
11	Bisabolol oxide B
12	Azulene
13	4-Hydroxycoumarine
14	6-Hydroxycoumarine
15	7-Hydroxycoumarine
16	Luteolin
17	Patuletin
18	Herniarin
19	Apigenine-7-O-glucoside
20	Apigenin-8-C-glucoside
21	alpha-Bisabolol acetate
22	Gallic acid
23	Vanillic acid-O-hexoside
24	Syringic acid
25	Caffeicacid-O-hexoside1
26	Neochlorogenic acid
27	Protocatechuic acid
28	Caffeicacid-O-hexoside-2
29	Homovanillicacid-O-hexoside-1
30	Caffeicacid-O-hexoside-3
31	p-Hydroxybenzoic acid
32	Chlorogenic acid
33	Coumaricacid-O-hexoside-1
34	m-Hydroxybenzoic acid
35	Cryptochlorogenic acid
36	Homovanillic acid
37	Caffeic acid
38	4-O-p-Coumaroylqunic acid
39	Coumaric acid-O-hexoside
40	Vanillic acid
41	p-Coumaric acid
42	Ferulic acid
43	Rutin
44	Kaempferol-3-O-rutinoside
45	Kaempferol-3-O-glucoside
46	Populnetin
47	Quercetin
48	Naringenin
49	Apigenin
50	Hesperetin

### 2.4 Animal model

Healthy 6-week-old male albino Balb/c mice were obtained from Beirut Arab University's animal facility. They were housed under standard laboratory conditions of light (12-hour light/dark cycle), temperature (22 ± 2 °C), and humidity with *ad libitum* access to standard mouse diet and tap water. Mice were left to acclimate with these conditions for one week before beginning the experiments. Experimental procedures were carried according to the approved guidelines of the Institutional Review Board (IRB) at Beirut Arab University code number 2018A-0033-S-M-0245.

### 2.5 Experimental design

**Figure 1:**
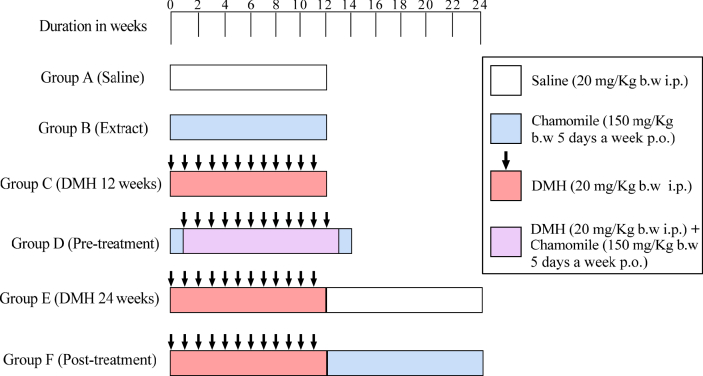
Schematic representation of treatment schedule.

Animals were randomly divided into six experimental groups of 6 mice each. The experimental protocol is shown in Table 2 and schematically represented in Figure 1.

**Table 2:** Experimental protocol.

Group A: Saline	Mice were intraperitoneally (i.p.) injected with saline once per week over a period of 12 weeks. Saline is the vehicle used to dissolve DMH.
Group B: Extract	Mice received *M. chamomilla* aqueous extract only at 150 mg/Kg/ b.w. by gavage (P.O.) 5 days/week for 12 weeks.
Group C: DMH 12 weeks	Mice received DMH dissolved in saline (20 mg/Kg b.w., i.p.) once per week over 12 weeks to induce colorectal cancer (CRC).
Group D: Pre-treatment	Mice were pre-treated with *M. chamomilla* extract (150 mg/Kg/ b.w.) starting 1 week before DMH injections and continued till 1 week after the final DMH exposure.
Group E: DMH 24 weeks	Mice received DMH for 12 weeks as in group C and left without any treatment for an additional 12 weeks.
Group F: Post-treatment	Mice received *M. chamomilla* extract (150 mg/Kg/ b.w.) starting 1 week after the twelfth DMH injection and continued over additional 12 weeks.

The concentration used to induce CRC was based on Gurley, Moser & Kemp [35]. The selected dose of chamomile extract was chosen on the basis of previously published reports and with reference to the average human daily intake [33, 36, 37, 38].

At the end of the treatment, mice were fasted overnight, then sacrificed and their livers were excised for further histological, biochemical and molecular analyses.

### 2.6 Tissue homogenization and protein quantification

Liver tissues were homogenized in phosphate buffered saline (PBS, pH 7.4) supplemented with 1 mM of the protease inhibitor PMSF at a ratio of 1 g per 5 mL of buffer. The homogenate was then centrifuged for 15 min at 15,000 rpm at 4 °C, and the supernatant was collected and stored at – 80 °C for later use.

Proteins concentration in all tissue homogenates were quantified according to the method of Lowry *et al.* using BSA (Bovine serum albumin) as a standard [39]. Each sample was run in triplicates. Absorbance of the developed color was assessed spectrophotometrically at 650 nm. Protein concentrations were deduced from the BSA standard curve.

### 2.7 Enzyme assays for alanine amino transferase (ALT) and aspartate amino transferase (AST)

ALT and AST activities in all tissue homogenates were measured using the GPT (ALT) colorimetric assay kit (cat# BEIS45-E, Spin-React®, Spain) and GOT (AST) assay colorimetric kit (cat# MDBEIS46-P, Spin-React®, Spain), respectively and as recommended by the manufacturer.

### 2.8 Quantification of Wnt signaling gene expression by RT-PCR

#### 2.8.1 RNA extraction and quantification

Total RNA was extracted from liver homogenates using the RNeasy Plus Mini Kit (catalog # 732-6820, QIAGEN®) according to manufacturer recommendations. In brief, 200 μL of liver tissue homogenate were lyzed in 400 μL of denaturing guanidine-thiocyanate–containing RLT buffer, which inactivates RNases and ensures intact RNA isolation. The lysate was then passed through a gDNA eliminator spin column to eliminate genomic DNA. Ethanol was also added to the lysate in order to create conditions that promote selective binding of RNA to the RNeasy column. The sample was then applied to the RNeasy spin column, where total RNA binds to the membrane and contaminants are efficiently washed away. High-quality RNA was finally eluted in 80 μL RNase-free water.

To check for the integrity of the eluted RNA, samples were electrophoretically separated on 1% agarose and visualized by UV illumination using ethidium bromide staining. RNAs appeared as two sharp bands corresponding to the 28S rRNA and 18 S rRNA. RNA was quantified through its absorbance which was measured at 260 nm. Its purity was assessed from the 260/280 absorbance ratio.

#### 2.8.2 Reverse transcription

RNA was transcribed using the QuantiTect® Reverse Transcription Kit (catalog # 205311, QIAGEN®) according to manufacturer recommendations. In brief, 1.0 μg of RNA samples were incubated in 3 μL gDNA Wipeout Buffer and 9 μL of RNase free water at 42 °C for 2 min to effectively remove contaminating genomic DNA in a total volume of 14 μL. After genomic DNA elimination, RNA samples were reverse transcribed using Quantiscript Reverse Transcriptase (1.5 μL), Quantiscript RT Buffer (6 μL), and RT Primer Mix (1.5 μL) in a final volume of 20 μL. The reaction took place at 42 °C for 15 min and the enzyme was then inactivated at 95 °C for 3 min. Finally, the cDNA obtained were stored at –80 °C for later use.

#### 2.8.3 RT-PCR

The expression of Wnt signaling genes were quantified by RT-PCR using QuantiFast® SYBR® Green PCR Kit (catalog # 204045, QIAGEN®). The amplification reaction was carried out at final volume of 10 μL containing 5 μL of 2x QuantiFast SYBR Green PCR Master Mix, 1 μL (1 μM) of each primer (forward and reverse), 2 μL of cDNA and 1 μL of RNase-free water. Cycling was performed as follows. First, a denaturation step at 95 ° C for 5 min, followed by 45 cycles of denaturation at 95 ° C for 10 s and annealing/extension at 60 ° C for 30s.

Forward (F) and reverse (R) sequences are shown in Table 3 along with expected product size to be amplified (bp).

**Table 3:** Sequences of forward and reverse primers used to amplify the selected genes.

Gene	Primer Sequence	Product size (bp)
GAPDH	**F**: 5′-TGGTGCTCAGTGTAGCCCAG-3′ **R**: 5′-GGACCTGACCTGCCGTCTAG-3′	111
Wnt5a	**F:** 5′-CTGGCAGGACTTTCTCAAGG-3′ **R:** 5′-CTCTAGCGTCCACGAACTCC-3′	395
GSK3β	**F:** 5′-TCCATTCCTTTGGAATCTGC-3′ **R:** 5′-CAATTCAGCCAACACACAGC-3	236
APC	**F:** 5′-TGGAAGTGTGAAAGCATTGATGGAATGTGC-3′ **R:** 5′-CCACATGCATTACTGACTATTGTCAAG-3′	348
β-Catenin	**F:** 5′-GCTGACCTGATGGAGTTGGA-3′ **R:** 5′-GCTACTTGCTCTTGCGTGAA-3′	227
Lef1	**F:** 5′-TGAGTGCACGCTAAAGGAGA-3′ **R:** 5′-ATAATTGTCTCGCGCTGACC-3′	160
Tcf4	**F:** 5′-CAAAGAAAGTCCGAAAAGTTCCT-3′ **R:** 5′-GGCGAGTCCCTGTTGTAGTC-3′	88
C-Myc	**F:** 5′-TAGTGCTGCATGAGGAGACA-3′ **R:** 5′-GGTTTGCCTCTTCTCCACAG-3′	104
Cyclin D1	**F:** 5′-GGCACCTGGATTGTTCTGTT-3′ **R:** 5′-CAGCTTGCTAGGGAACTTGG-3′	232

**F**: Forward set; **R**: Reverse set; **bp** = base pair.

Gene expression was measured by comparative threshold cycle (Ct) method using glyceraldehyde-3 phosphate dehydrogenase (GAPDH) as a reference gene. For each gene, the mean Ct (mCt) values were determined. ΔCt value was determined as the difference between the Ct of gene of interest and the Ct of GAPDH gene. The relative quantity of gene of interest expression compared to GAPDH gene was calculated applying the gene dosage ratio formula (GDR = 2^−ΔΔCt^) where:

ΔΔCt = (mCt gene of interest − mCt GAPDH) control sample − (mCt gene of interest − mCt GAPDH) test sample.

### 2.9 Quantification of pro-inflammatory enzymes

The level of COX-2 was measured using SimpleStep ELISA® kit (abcam®, MA, USA) according to manufacturer recommendations. The activity of iNOS was measured using Nitric Oxide Synthase Assay Kit (Abnova, CA, USA) according to manufacturer recommendations.

### 2.10 Statistical analysis

All statistical analyses were performed using Microsoft Excel, and they are shown as mean with standard deviations. Statistical significance was assessed using One-way ANOVA test followed by Tukey test. Graphs were drawn using GraphPad prism software and statistical significance was reported with a p-value < 0.05 considered as significant. Results with ∗∗∗∗ indicate the significance at *P* < 0.0001, ∗∗∗ at *P* < 0.001, ∗∗ at *P* < 0.01, and ∗ at *P* < 0.05.

## 3 Results

### 3.1 Profile of the aqueous chamomile extract

Out of 50 polyphenols and flavonoids screened for, 28 polyphenols were detected in the aqueous extract of the chamomile via direct injection into the MS, whereby detection was confirmed through a signal intensity in excess of e^1^ (Table 4). Among the detected compounds, the highest signal intensity was in the order of e^4^ and corresponds to herniarin, chlorogenic acid and ferulic acid. Signals in the order of e^3^ were observed for alpha-bisabolol, chamazulene, bisabolol oxide B, apigenin-8-C-glucoside, protocatechuic acid, p-hydroxybenzoic acid, homovanillic acid, caffeic acid, vanillic acid, p-coumaric acid, kaempferol-3-O-glucoside, and naringenin.

**Table 4:** List of polyphenols and flavonoids present in the chamomile aqueous extract as detected via LC-MS/MS. (CE stands for collision energy, Pos ESI stands for Positive Electrospray Ionization, Neg ESI stands for Negative Electrospray Ionization, and m/z represents mass divided by charge number).

#	Compound	Ionisation	Exact Mass	m/z	Ions	CE
1	alpha-Bisabolol	pos ESI	222.198	223.206	205.19, 69.07	20
2	Chamazulene	pos ESI	184.125	185	169	5
3	alpha-Pinene	pos ESI	136.125	137.13	121.1, 105.07	40
4	3-Epinobilin	pos ESI	346.178	347.1858	247.13, 83.04	20
5	Bisabolol oxide B	pos ESI	238.193	239.2	221.19, 81.07	20
6	4-Hydroxycoumarine	pos ESI	162.032	163.0395	51.0235, 121.0290, 163.0395	40
7	7-Hydroxycoumarine	pos ESI	162.032	163.0395	119.0497, 145.0290	40
8	Luteolin	pos ESI	286.048	287.0556	153.0188, 109.0290, 213.0552, 269.0450	40
9	Herniarin	pos ESI	176.047	177.0552	77.0391, 121.0290, 103.0548, 133.0653, 147.0446	40
10	Apigenin-8C-glucoside	pos ESI	432.106	433.1129	415.1, 397.1, 367.1 (10 EV)	5
11	Gallic acid	pos ESI	170.022	171.0293	153.0188, 125.0239	20
12	Syringic acid	pos ESI	198.053	199.0606	181.0501	20
13	Protocatechuicacid	pos ESI	154.027	155.0344	109.0290, 137.0239	20
14	p-Hydroxybenzoicacid	pos ESI	138.032	139.0395	121.0290, 95.0503	20
15	Chlorogenicacid	pos ESI	354.095	355.1029	163.0395, 337.0923, 193. 0712, 175.0606	20
16	m-Hydroxybenzoicacid	pos ESI	138.032	139.0395	93.034	20
17	Homovanillicacid	pos ESI	182.058	183.0657	137.0603, 165.0552	20
18	Caffeicacid	pos ESI	180.042	181.0438	135.0446, 163.0395	20
19	Vanillicacid	pos ESI	168.042	169.0501	151.0395, 123.0446	20
20	p-Coumaricacid	pos ESI	164.047	165.0552	91.0544, 147.0446, 119.0497 (30 EV)	20
21	Ferulicacid	pos ESI	194.058	195.0657	177.0552, 149.0603	20
22	Kaempferol-3-O-glucoside	neg ESI	448.101	447.0934	284.0237, 255.0294, 227.0341	40
23	Rosmarinicacid	neg ESI	360.085	359.0772	161.0240, 359.0767, 197.0454, 135.0709	40
24	Populnetin	neg ESI	286.048	285.0415	164.9985, 255.0296, 227.0357, 117.0346	20
25	Quercetin	neg ESI	302.043	301.0373	151.0013, 178.9964, 271.0250	20
26	Naringenin	neg ESI	272.068	271.0606	135.0082, 119.0497, 151.0031, 93.0340, 109.0290, 83.0133	40
27	Apigenin	neg ESI	270.053	269.052	117.038, 151.0080	30
28	Hesperetin	neg ESI	302.079	301.0722	136.0169, 151.0042, 164.0118, 285.0403	40

### 3.2 Liver-specific injury enzymes

**Figure 2:**
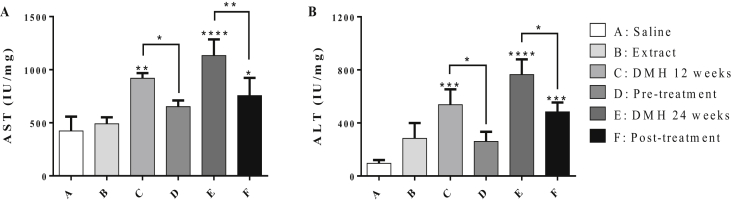
Effect of chamomile on the levels of AST (A) and ALT (B) in liver tissues of treated mice. Data represented are the mean of three determinations ±SD. (∗), (∗∗), (∗∗∗), and (∗∗∗∗) correspond to *P* < 0.05, 0.01, 0.001, and 0.0001 respectively.

Figure 2 shows the activities of AST and ALT in serum for all groups. DMH induced a significant increase in AST and ALT levels (groups C and E) compared to control group (Group A). Pre-treatment of DMH-injected mice with chamomile (Group D) significantly minimized the liver damage. Significant reduction in the levels of AST and ALT (~30% and 52% respectively, *P* < 0.05) was obtained as compared to Group C. Likewise, chamomile post-treatment (Group F) significantly reduced the levels of AST and ALT by ~33% (*P* < 0.01) and 37% (*P* < 0.05), respectively, compared to the untreated mice in Group E.

### 3.3 Gene expression levels of Wnt pathway regulators

#### 3.3.1 Oncogenes: Wnt5a and β-catenin

**Figure 3:**
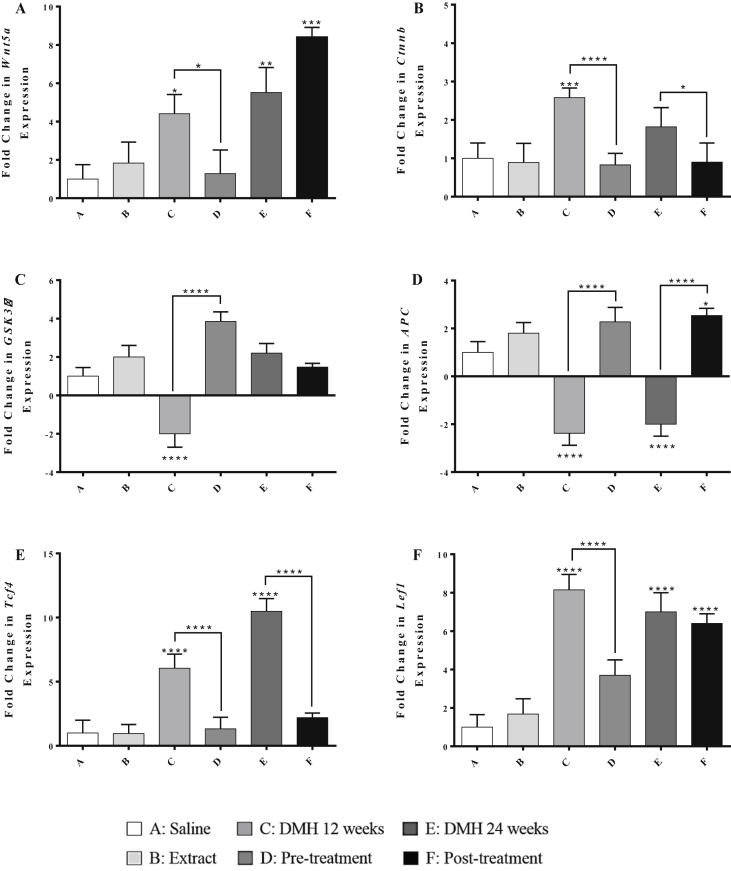
Effect of chamomile on the expression levels of Wnt5a (A), β-catenin (B), GSK3β (C), APC (D), Tcf4 (E), and Lef1 (F) genes in liver tissues of treated mice. Expression levels of treated and control groups were normalized to their respective GAPDH. Fold expression was determined relative to the control. All bars represent mean of three determinations ±SD. (∗), (∗∗), (∗∗∗), and (∗∗∗∗) on bars and on lines drawn upwards, that represent inter-categorical statistical significance, correspond to *P* < 0.05, <0.01, <0.001, and <0.0001 respectively.

As shown in Figure 3, Panels A and B, DMH administration for 12 weeks (Group C) induced significant upregulations in the expression of Wnt5a by 3.4 folds (*P* < 0.05) and β-catenin by 1.58 folds (*P* < 0.001) compared to the control group. Similarly, DMH-treated groups for 24 weeks (Group E) showed a significant upregulation in Wnt5a gene by 4.5 folds (*P* < 0.01) compared to the control group. However, chamomile pre-treatment of DMH-injected mice (Group D) significantly downregulated the expression level of Wnt5a (3.2-fold decrease, *P* < 0.05) and β-catenin (1.9-fold decrease, *P* < 0.0001) genes, compared to those receiving the carcinogen only (Group C). Chamomile post-treatment of DMH-injected mice (Group F) significantly downregulated the expression level of β-catenin (0.9-fold decrease, *P* < 0.0001) compared to Group E.

#### 3.3.2 Tumor suppressor genes: GSK3β and APC

In Panels C and D of Figure 3, DMH administration for 12 weeks (Group C) showed decreased expression levels of GSK3β and APC genes by 3 and 3.38 folds (*P* < 0.0001) respectively, compared to the normal control (Group A). However, pre-treated mice with chamomile extract (Group D) significantly increased the expression level of GSK3β and APC gene by 5.85 and 4.66 folds (*P* < 0.0001) respectively, compared to Group C. Chamomile, as a post-treatment (Group F), exerted a significant upregulation in the expression levels of APC gene by 4.54 folds in the liver (*P* < 0.0001) compared to group E.

#### 3.3.3 Transcription factors: Tcf4 and Lef1

DMH administration for 12 weeks (Group C) and 24 weeks (Group E) induced significant upregulation in the expression of Tcf4 gene by 5 folds and 9.4 folds (*P* < 0.0001) respectively, compared to the control group as illustrated in Panels E and F of Figure 3. These alterations were modulated by chamomile pre- and post-treatments that induced significant downregulations in Tcf4 level by 4.7 folds and 7.3 folds (*P* < 0.0001) respectively.

As for the Lef1 gene expression, DMH administration for 12 weeks upregulated the level of Lef1 by 2 folds, and this effect was significantly reversed by chamomile pre-treatment which downregulated the expression level of this gene by 2.4 folds (*P* < 0.0001).

### 3.4 Gene expression levels of cell cycle regulators

**Figure 4:**
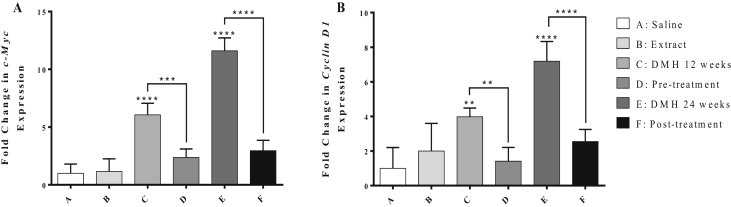
Effect of chamomile on the expression levels of c-Myc (A) and Cyclin D1 (B) genes in liver tissues of treated mice. Expression levels of treated and control groups were normalized to their respective GAPDH. Fold expression was determined relative to the control. All bars represent mean of three determinations ±SD. (∗), (∗∗), (∗∗∗), and (∗∗∗∗) on bars and on lines drawn upwards, that represent inter-categorical statistical significance, correspond to *P* < 0.05, <0.01, <0.001, and <0.0001 respectively.

As shown in Figure 4, DMH-treatment for 12 and 24 weeks significantly upregulated the expression level of c-Myc gene by 5 and 10.6 folds (*P* < 0.0001) respectively, as well as the levels of Cyclin D1 gene by 2.9 (*P* < 0.01) and 6.2 folds (*P* < 0.0001) respectively, compared to the control. However, pre-treatment with chamomile extract caused a significant downregulation in the expression level of c-Myc and Cyclin D1 by 3.7 folds (*P* < 0.001) and 2.5 folds (*P* < 0.01) respectively, compared to group D. Similarly, chamomile as a post-treatment caused significant downregulation in the expression level of c-Myc gene by 8 folds and Cyclin D1 gene by 4.6 folds (*P* < 0.0001) respectively compared to group E.

### 3.5 COX-2 level and iNOS activity

**Figure 5:**
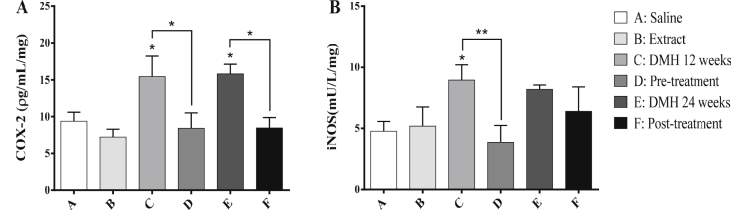
Effect of chamomile on the level of COX-2 (A) and activity of iNOS (B) in liver tissues of treated mice. Data are represented as mean ± SD. (∗), (∗∗), (∗∗∗), and (∗∗∗∗) correspond to *P* < 0.05, 0.01, 0.001, and 0.0001 respectively.

DMH induced a significant elevation in COX-2 level by 40% and 40.7% (*P* < 0.05) after 12 and 24 weeks respectively, compared to the control group as shown in Figure 5. Chamomile pre and post-treatments (Groups D and F) caused significant decrease in COX-2 level by 45.5% and 46.5% (*P* < 0.05) respectively, compared to groups C and E. As for iNOS, chamomile pre-treatment of DMH-injected mice (Group D) led to a significant decrease in its activity by 56.7% (*P* < 0.05) compared to group C.

## 4 Discussion

CRC occurs as a result of many multistep processes that lead to the accumulation of mutations in tumor-suppressor genes and oncogenes [40]. Recently, the role of oxy-radicals during different stages of carcinogenesis has been reported whereby ROS play crucial roles in the metabolic activation of carcinogens. DMH acts as a potent necrogenic hepatic carcinogen that mainly alkylates the hepatocellular DNA leading to hepatocarcinoma [41]. In the liver, DMH is metabolized into the active electrophile, the carbonium ion that elicits DNA mutations and oxidative stress [11]. Thus, this carcinogen has been commonly used as a model to evaluate the effect of therapeutic agents on hepatic molecular and enzymatic alterations that occur during different stages of carcinogenesis [42,43]. Since the conventional treatment methods of CRC share common serious side effects, there is an enduring popularity of herbal medicine mainly due to the ability of herbs to function slowly, effectively and with minimal toxic side effects. Therefore, in this work we aimed to assess the hepatoprotective effects of *M. chamomilla* extract during an intermediate DMH-induced mouse model of CRC, taking into consideration the activation of Wnt signaling pathway and inflammation.

At the biochemical level, DMH-induced hepatotoxicity was evidenced by significant elevations in the levels of serum ALT and AST that are attributed to liver injury. The increments of serum ALT and AST activities are usually due to their leakage from liver cytosol into blood in cases of necrosis or membrane damage, making these enzyme potent markers of hepatic damage [44]. The present results demonstrated that chamomile extract significantly minimized liver injury via decreasing the levels of AST and ALT. Moreover, our results are in agreement with a study by Mannaa *et al.* [45] that reported the hepatoprotective effects of chamomile flowers extract against azathioprine-induced liver injury through modulating the levels of ALT and AST enzymes. Similarly, AST and ALT levels were reduced after *Matricaria chamomilla* treatment in streptozotocin-induced diabetic rats [46]. Likewise, our results are consistent with previous studies where chamomile tea modulated the activity of hepatic cytochrome P450 [47], exerted hepatoprotective activity against paracetamol-induced liver damage in albino rats [29], and reduced hepatic damage and oxidative stress by positively modifying several enzyme systems including AST and ALT in carbon tetrachloride treated rats [48].

At the molecular level, since DMH metabolites were shown to alkylate DNA causing mutations in Apc and β-catenin gene which are key players of the Wnt pathway [11, 12, 13, 14, 15], targeting this signaling pathway reveals insights into novel chemopreventive strategies. In our study, we investigated the altered Wnt signaling through assessing the gene expressions of: Wnt5a, APC, GSK3β, β-catenin, Tcf4 and Lef1, as well as c-Myc and Cyclin D1. Our results showed that this pathway was activated in the liver tissues of DMH mice. Interestingly, pre-treatment with chamomile extract significantly modulated the alterations in this pathway, was more effective than post-treatment, and led to a reduction in hepatic cell proliferation.

It is quite clear from the obtained findings that a significant hepatic damage has been elicited, in the form of elevated AST and ALT levels and activated Wnt signaling pathway, after DMH treatment. While gene changes reflect long term damage attributed to DNA alkylating ability of DMH metabolites [11], chemical changes reflect the continuous hepatic damage caused by DMH metabolites as well as from ROS such as H_2_O_2_ released from colonic tumors [49]. A study by Zatrowski and Nathan suggested that tumor cells can further produce substantial amount of H_2_O_2_ into the circulation which can then reach the liver for detoxification [50]. Therefore, the liver damage induced by DMH in the present study, especially in post-initiation groups E & F, could be attributed to the excessive generation of H_2_O_2_ – by the DMH-induced colon tumors – that has been transferred to the liver for detoxification. Moreover, Burton *et al.* showed that cancer cells tend to protect themselves and grow by releasing products of lipid peroxidation which also explains the continuous liver damage for weeks after DMH exposure [51].

The putative hepatoprotective and anti-proliferative activities of chamomile extract might be explained at least in part by the well-documented activities of its bioactive compounds. Phenolic compounds, such as flavonoids, tannins, coumarins, lignans, and quinones, are secondary compounds known for their hepatoprotective, antitumor and anti-inflammatory activities [52]. In the present study, the phytochemical screening revealed the presence of flavonoids: quercetin, apigenin, luteolin, rosmarinic acid, caffeic acid, and gallic acid in the aqueous extract of *M. chamomilla*. The hepatoprotective and anti-proliferative effects of these flavonoids have been extensively studied in the literature. For example, apigenin has been shown to possess cancer-preventive and anti-cancer activities against different types of cancers via inhibiting the Wnt/β-catenin signaling [53,54]. In addition, a recent study by Chiang *et al.* showed that apigenin exerts anti-hepatoma activities via an apoptotic mechanism that is mediated through the p53-dependent pathway and the induction of p21 expression leading to cell cycle arrest in G2/M phase [55]. Luteolin, another potent flavonoid found in our extract, has been studied for its ability to exert an anticancer activity against HepG2 cells by inducing apoptosis, causing G1 cell cycle arrest, and regulating the expression levels of p21, Bax and caspase-3 [56]. In CRC, it exerts its antitumor effects via inhibiting DMH-induced cell proliferation that involves the Wnt/β-catenin pathway [57]. Also, luteolin was shown to exert potent curative ability through decreasing the activity of different liver enzymes including AST and ALT against hepatocellular carcinoma in rats [58], and against acetaminophen-induced liver injury in mice [59]. Other phytochemicals such as quercetin, rosmarinic acid, caffeic acid, and gallic acid have been well-studied in the literature for their hepatoprotective effects against chemical-induced hepatotoxicity in rodents [60, 61, 62, 63].

Moreover, cancers involve serious complications of inflammation, where the enzymes COX-2 and iNOS provide a link between inflammation and carcinogenesis [64]. The Wnt/β-catenin pathway is known to up-regulate the expression of COX-2 [65]. Additionally, iNOS can stimulate and enhance COX-2 activity through a transcriptional pathway mediated by Wnt/β-catenin [66]. Therefore, based on our signaling results, we went further to investigate the effect of chamomile on COX-2 and iNOS. Our data showed that the pre-treatment of DMH mice with chamomile extract resulted in significant downregulation of COX-2 levels and iNOS activities compared to mice receiving DMH alone. Likewise, our results demonstrate that chamomile acts as COX-2 and iNOS inhibitor, and this is consistent with previous studies that showed that chamomile extract acts as COX-2 inhibitor during gastric damage [67] and iNOS inhibitor in RAW 264.7 macrophages [68]. Moreover, studies by Pandurangan *et al.* showed that luteolin – a major flavonoid found in chamomile – decreased the expressions of iNOS and COX-2 in AOM-induced CRC in mice [69] and induced growth arrest in colon cancer cells via the Wnt/β-catenin/GSK-3β signaling [70].

More importantly, our findings are in concomitance with a study by El Joumaa *et al.* [33] who showed that chamomile extract suppressed CRC incidence and progression in DMH-induced mouse model of colorectal carcinogenesis. In that model, chamomile extract inhibited tumor incidence and multiplication, downregulated the Wnt signaling pathway, and mitigated inflammation by modulating the levels of the pro-inflammatory enzymes COX-2 and iNOS in the colonic tissues of DMH-treated mice. Our results extended that work, corroborated the protective effect of chamomile, and provided better understanding of its activities in the liver, whereby it exerted protective effects against DMH-induced damage and carcinogenicity in the liver and subsequently in the colon as proven earlier.

In comparison with other herbs and dietary agents, similar effects were observed in a study by Devasena *et al.* [49] where a curcumin analog ameliorated the DMH-induced hepatic oxidative stress during colon carcinogenesis. Other studies by Sengottuvelan *et al.* [71] and Jrah-Harzallah *et al.* [72] showed the modulatory influence of dietary resveratrol and thymoquinone, respectively, during different phases of DMH-induced hepatic injury and oxidative stress during CRC. Moreover, our results are consistent with a study by Sharma and Sharma which indicated a chemoprotective role of Triphala against DMH-induced carcinogenic damage to mouse livers [73].

In conclusion, *M. chamomilla* extract is a natural dietary agent with profound biological and pharmacological properties that ameliorate the hepatic damage induced by the carcinogen DMH. Further investigations are needed to provide assessments of oxidative stress and DNA damage caused by DMH. Also, our data open up future work for additivity and/or synergism of chamomile extract with Wnt/COX-2/iNOS inhibitors for the development of more potent therapies with minimal side effect, and identification of the active molecules responsible for antiproliferative and hepatoprotective activities.

## Declarations

### Author contribution statement

J. Borjac: Conceived and designed the experiments; Analyzed and interpreted the data; Wrote the paper.

S. Shebbo and R. Kawash: Performed the experiments; Analyzed and interpreted the data; Contributed reagents, materials, analysis tools or data.

M. El Joumaa: Performed the experiments; Analyzed and interpreted the data; Contributed reagents, materials, analysis tools or data; Wrote the paper.

### Funding statement

This research did not receive any specific grant from funding agencies in the public, commercial, or not-for-profit sectors.

### Competing interest statement

The authors declare no conflict of interest.

### Additional information

No additional information is available for this paper.
